# Biogeographic congruency among bacterial communities from terrestrial sulfidic springs

**DOI:** 10.3389/fmicb.2014.00473

**Published:** 2014-09-08

**Authors:** Brendan Headd, Annette S. Engel

**Affiliations:** Department of Earth and Planetary Sciences, University of TennesseeKnoxville, TN, USA

**Keywords:** sulfur-oxidizing bacteria, microbiome, biogeography, *Epsilonproteobacteria*, *Gammaproteobacteria*, 16S rRNA

## Abstract

Terrestrial sulfidic springs support diverse microbial communities by serving as stable conduits for geochemically diverse and nutrient-rich subsurface waters. Microorganisms that colonize terrestrial springs likely originate from groundwater, but may also be sourced from the surface. As such, the biogeographic distribution of microbial communities inhabiting sulfidic springs should be controlled by a combination of spring geochemistry and surface and subsurface transport mechanisms, and not necessarily geographic proximity to other springs. We examined the bacterial diversity of seven springs to test the hypothesis that occurrence of taxonomically similar microbes, important to the sulfur cycle, at each spring is controlled by geochemistry. Complementary Sanger sequencing and 454 pyrosequencing of 16S rRNA genes retrieved five proteobacterial classes, and Bacteroidetes, Chlorobi, Chloroflexi, and Firmicutes phyla from all springs, which suggested the potential for a core sulfidic spring microbiome. Among the putative sulfide-oxidizing groups (*Epsilonproteobacteria* and *Gammaproteobacteria*), up to 83% of the sequences from geochemically similar springs clustered together. Abundant populations of *Hydrogenimonas-like* or *Sulfurovum-like* spp. (*Epsilonproteobacteria*) occurred with abundant *Thiothrix* and *Thiofaba* spp. (*Gammaproteobacteria*), but *Arcobacter-like* and *Sulfurimonas* spp. (*Epsilonproteobacteria*) occurred with less abundant gammaproteobacterial populations. These distribution patterns confirmed that geochemistry rather than biogeography regulates bacterial dominance at each spring. Potential biogeographic controls were related to paleogeologic sedimentation patterns that could control long-term microbial transport mechanisms that link surface and subsurface environments. Knowing the composition of a core sulfidic spring microbial community could provide a way to monitor diversity changes if a system is threatened by anthropogenic processes or climate change.

## Introduction

Dispersion (Martiny et al., 2006; Ramette and Tiedje, 2006; Telford et al., 2006) and transport due to wind (Smith et al., 2013), water (Sinton et al., 1997; Crump et al., 2012), and biology (Grossart et al., 2010) distribute microbes into different environments. But, genetic regulation of electron donor and acceptor utilization, based on availability from an environment, controls microbial metabolism, growth, and reproduction (e.g., Green et al., 2008). Consequently, the dominance of specific microbial groups in a given habitat should correspond to when and where metabolic requirements are met, while also reflecting past and present surface and subsurface transport and dispersion mechanisms (including transport from the surface to the subsurface, or vice versa).

In terrestrial settings, a spring is a hydrological feature where an aquifer intersects the earth's surface. Although rock type (e.g., sandstone, limestone) and properties (e.g., fractured, karstified), as well as hydrostatic pressure and flow dynamics, influence where a spring discharges at the surface (Manga, 2001; Pitts and Alfaro, 2001; Cantonati et al., 2012), the position of a spring and its water composition can be nearly constant over time due to stable aquifer hydrogeochemical conditions (Prescott and Habermehl, 2008). Springs are often colonized by microorganisms that form extensive microbial mats with metabolically diverse community compositions (Camacho et al., 2005) and whose members may have originated from the subsurface (Tin et al., 2011). Many earlier studies have focused on characterizing microbial populations at single spring sites and correlating some microbial groups to spring geochemistry (Skirnisdottir et al., 2000; Elshahed et al., 2003; Perreault et al., 2007; Chaudhary et al., 2009). A few studies attempt to correlate microbial genes (Zhang et al., 2008) and populations across multiple springs separated by varying geographic distances (Rudolph et al., 2004; Porter and Engel, 2008). But, there has been limited work to correlate present-day microbial communities separated by large geographic distances and differing in geologic processes (and geologic time) that may be affected by spring geochemistry (e.g., Bahl et al., 2011).

We focused our investigation on terrestrial sulfidic springs because these springs could allow us to test hypotheses related to whether or not geochemical conditions, established by hydrogeological connectivity (either past or present) across local to regional geographic distances, control microbial community compositions, as well as if a core sulfidic spring microbiome could be defined. We hypothesized that geochemically similar springs, regardless of geographic distance, have shared microbial communities, a so-called core microbiome. To evaluate a core microbiome, we used taxonomic presence vs. absence data of 16S rRNA genes that formed shared operational taxonomic units (OTUs). Dominant microbial populations in the springs were putative sulfide-oxidizing bacteria and many groups were shared among geographically separated springs with distinct geochemical conditions, which suggested a core microbiome exists among springs of similar geochemistry. Long-term geologic histories likely influenced transport and discharge of microorganisms at each spring, resulting in similar distribution patterns for some epsilonproteobacterial and gammaproteobacterial groups. The widespread distribution of these groups across a range of geochemical conditions and geographic distances may be due to their ability to withstand widely variable geochemical conditions over long periods of time. From a predictive standpoint, knowing the composition of a common sulfidic spring microbial community may provide resource managers a way to monitor diversity changes in a system if threatened by anthropogenic processes or climate change (Lutz, 2004; Barquín and Scarsbrook, 2008; Cantonati et al., 2012) because sulfidic springs are presently or have been exploited for recreational or mineral water resource needs (Williams et al., 1996; Spivack, 1998; Erfurt-Cooper and Cooper, 2009).

## Materials and methods

### Sample acquisition and site descriptions

**Figure 1:**
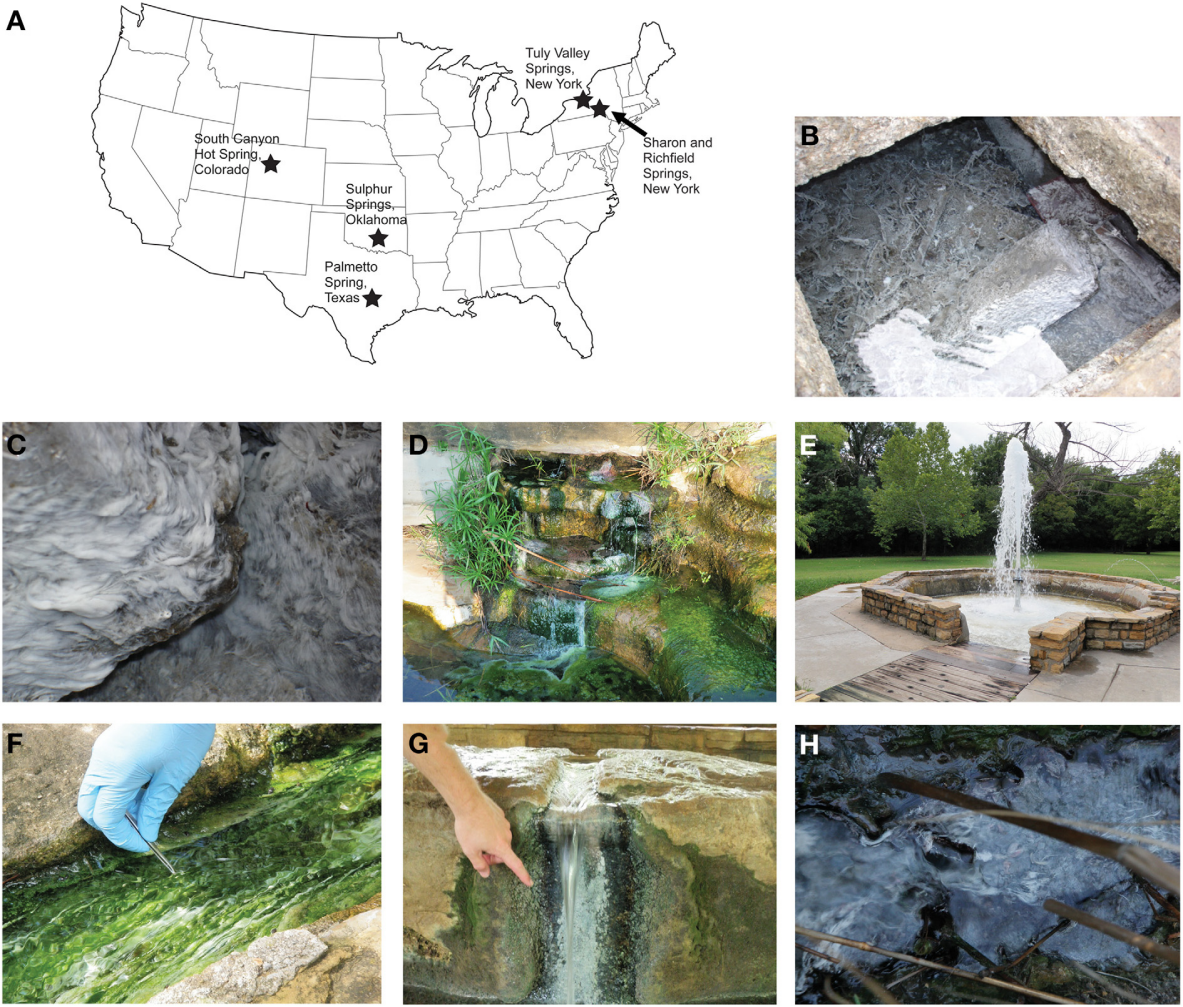
**(A)** General locations of terrestrial sulfidic springs where microbial mats were sampled in this study. Map graphic modified from http://www.pageresource.com/clipart. Representative photographs from each sampling location: **(B)** looking into the cistern at Sharon Springs (photograph is ~1 m across); **(C)** close-up of microbial mats colonizing concrete basin at Richfield Springs (photograph is ~10 cm across); **(D)** concrete wall at Palmetto Spring; **(E)** Vendome well fountain at Sulphur Springs; **(F)** outflow channel from Hillside Spring at Sulphur Springs, **(G)** Pavilion Spring at Sulphur Springs; **(H)** close-up of filaments mixed with mud and vegetation at South Canyon Hot Spring (photograph is ~10 cm across).

Seven continental sulfidic springs in the USA were sampled from 2008–2010 to span a range of geochemical conditions, bedrock geology, and hydrological settings that overlapped or supplemented previously studied springs (e.g., Rudolph et al., 2004; Porter and Engel, 2008; Rossmassler et al., 2012) (Figure 1A, Table 1). At each spring, several grams of white, filamentous microbial mat material were collected aseptically and placed into replicate 2 ml cryogenic vials that were stored on ice during transport. All other colored microbial mats (e.g., green, brown, gray) or morphologically different material were avoided, as a way to control the study. For the New York, Texas, and Colorado springs, water temperature, conductivity, and pH were measured in the field using standard electrode methods, as previously described (Rossmassler et al., 2012). Geochemical measurements were obtained for the Tully Valley (TV) springs in New York in November 2012. Total dissolved sulfide and dissolved oxygen were measured in the field using the Methylene Blue and Rhodazine D CHEMetrics^®^ (Calverton, Virginia, USA) colorimetric methods. Data reported in the literature were used for springs sampled in Oklahoma (Christenson et al., 2009a).

**Table 1:** **Geographic and geochemical details from springs sampled in this study**.

**Sample site**	**Location**	**Distance to other springs (km)**							**Temp °C**	**pH**	**Conductivity μS/cm**	**Dissolved sulfide μmol/L**	**Dissolved oxygen μmol/L**
		**S**	**R**	**TV1**	**TV2**	**OK**	**P**	**SCHS**					
Sharon Springs (S)	Schoharie County, NY	–	30	125	125	2142	2518	2753	8.50	7.09	2463	94.19	16.25
Richfield Springs (R)	Ostego County, NY		–	95	95	2116	2496	2721	7.50	7.07	2068	142.31	29.68
Tully Valley 1^*^ (TV1)	Onondaga County, NY			–	0.002	2026	2414	2628	11.70	8.35	28750	588.30	3.12
Tully Valley 2^*^ (TV2)	Onondaga County, NY				–	2026	2414	2628	11.40	9.75	32800	124.70	3.12
Vendrome Well, Sulphur Springs^**^ (OK)	Murray County, OK					–	549	1083	19.91	7.27	2100	40.51	215.931
Palmetto Springs (P)	Gonzales County, TX						–	1426	28.60	8.75	647	94.48	16.25
South Canyon Hot Spring (SCHS)	Garfield County, CO							–	45.30	7.18	1775	40.49	16.56

* *Geochemical data obtained in December 2012*.

** *Geochemical data from Christenson et al. (2009a)*.

The springs at Sharon Springs, New York, are publically accessible and were once part of a historically active resort. The springs discharge at the contact between the Silurian Brayman Shale and the overlying dolomitic Cobleskill Limestone (Berdan, 1950). Water and white filamentous microbial mats were collected from concrete and stone basins from the drinking spring and cistern spring (Figure 1B) at the White Sulphur Spring site. Richfield Springs is ~30 km west of Sharon Springs, also accessible to the public, and the geology and hydrology are similar to that of Sharon Springs. Water and thin white filamentous mats were collected from flowing water that filled a concrete and stone basin made at the spring head (Figure 1C). Sharon and Richfield springs have been previously sampled and described by Porter and Engel (2008) and Rossmassler et al. (2012).

TV springs (~25 in total) are located on private property, south of the town of Lafayette, New York. The TV area is underlain by significant evaporite deposits (Kappel, 2000). The springs originated in April 1993, following a landside caused by above normal precipitation that increased subsurface flow that led to subsidence of unconsolidated glacial deposits that overlay Devonian and Silurian dolomites, limestones, shales, and evaporites (Getchell, 1995; Pair et al., 2000). The springs have variable geochemical compositions, with some amount of mixing from an upper freshwater aquifer in unconsolidated glacial material and a lower, brackish and sulfidic aquifer in the bedrock (Kantrowitz, 1970; Kappel et al., 1996). The springs are ~95 km west of Richfield Springs, and the general geologic history is similar to that of Sharon and Richfield Springs area (Hackett et al., 2009). Two sampled springs, TV1 and TV2, were separated by several meters and both discharged into small, irregularly shaped (and sized) natural pools surrounded by thick, tall grassy vegetation that served as substrate for thin, white films and filaments to attach.

Palmetto Spring is located in Palmetto State Park adjacent to the San Marcos River in Texas. Several sulfidic springs discharge within the park boundaries, and the sampled spring, designated as the “Artesian Well and Pond” by the park, discharges from a man-made concrete catchment that flows into a pond. Thick, white microbial mats were collected from coatings on concrete and floating in the pool (Figure 1D). Some green material associated with the white mats was also collected because it was difficult to avoid contact. The source of the springs is not known, but stratigraphic data prepared by the Texas Water Development Board indicate the source is likely the fresh to slightly saline sandstone aquifer of the Eocene Carrizo-Wilcox Group (Shafer, 1965; Hutchinson et al., 2009). The Carrizo-Wilcox Group sediments were derived from eroded rocks from the central and southern Rocky Mountains, western North America (to include Oklahoma and northern and western Texas), and northern Mexico (Mackey et al., 2012).

Sulphur Springs is a public park located in the Chickasaw National Recreation Area near the town of Sulphur, Oklahoma. The park is underlain by aquifers of the Cambrian to Ordovician Arbuckle and Simpson Groups, which are comprised of limestones, dolostones, and interbedded sandstones along the northern edge of the Arbuckle Mountains (Hanson and Cates, 1994). Much of the Arbuckle Mountains were eroded away during the late Paleozoic and the sediments were transported toward and deposited in western Oklahoma and northern and western Texas (Moore and Plummer, 1922; Jones and Hentz, 1988; Johnson, 2008). As many as 33 freshwater and mineral springs have been documented, although many have stopped flowing, were combined into single springs that discharge into creeks, or modified with pipes that divert natural spring flow into decorative stone and concrete catchments (Hanson and Cates, 1994). One well and two springs were sampled for this study. Vendome Well is an artesian well that emanates from a pipe that sprays water into the air (Figure 1E), similar to a fountain, and thick, white filamentous mats coating the well water catchment were collected. Well water is a mixture of 99% freshwater and 1% brine from the Arbuckle-Simpson Aquifer (Christenson et al., 2009a). Sparse white filaments intermixed by thicker green mats were collected at Hillside Spring (Figure 1F). Thin, white, patchy filamentous mats growing within the concrete basin were collected at Pavilion Spring (Figure 1G), which has a man-made cover and no direct input of sunlight, vegetation, or sediment. Both Hillside and Pavilion Springs are believed to be sourced from the same waters as the Vendome Well (Christenson et al., 2009b).

South Canyon Hot Spring (SCHS) is a publically accessible spring located ~8 km from the town of Glenwood Springs, Colorado. The spring emanates from Holocene and late Pleistocene alluvial deposits that overly the Cretaceous Mancos Shale and Dakota Sandstone (Bryant et al., 2002), although the source of the water is unknown. Spring water seeps from several orifices in the alluvium and coalesces into a pool surrounded by vegetation (Figure 1H). Water and patchy, thin white filaments growing between grassy vegetation within a small channel were collected. SCHS starkly contrasts the sulfidic springs in nearby Glenwood Springs that have much higher salinity due to dissolution gypsum and anhydrite from the Pennsylvanian Eagle Valley Evaporite (Bryant et al., 2002). While the current drainage of the area is toward the west/southwest, in the past it may have been more southerly and easterly (Mackey et al., 2012). SCHS was also sampled by Rossmassler et al. (2012).

### DNA extraction, PCR amplification, and cloning

Total nucleic acids were extracted from microbial mat samples using the following protocol: Approximately 2 g of each of the environmental samples were placed into three different microcentrifuge tubes containing extraction buffer comprised of 100 mM Tris, 100 mM Na-EDTA, 100 mM Na-phosphate, 1.5 M NaCl, and 1% CTAB; samples were vortexed and placed in a 37°C water bath for 1 hour; 20 μl of proteinase K (20 mg/ml) and 20 μl lysozyme (20 mg/ml) were added to the tubes and a freeze-thaw series (three times at –20°C) was used to aid in disruption of the mat structure; samples were incubated at 55°C overnight to digest cellular material; RNase was added to the digests and the tubes were incubated at 37°C for 1 hour; proteins were precipitated in 7.5 M ammonium acetate; nucleic acids were precipitated in isopropanol for 1 hour and then washed in 70% ethanol and resuspended in 50 μl of TE Buffer.

Nearly full-length 16S rRNA genes were amplified from samples taken from each site using the 8F (forward, 5′-AGAGYYYGATYMTGGCTCAG −3′) and 1510R (reverse, 5′-TACGGYTACCTTGTTACGACTT-3′) primers, according to the protocol described by Lane (1991), with 5PRIME Perfect Taq polymerase and 25–30 ng/ul final concentration of nucleic acids. Amplification was performed under the following conditions for 30 cycles: denaturation at 94°C for 1 min, primer annealing at 50°C for 1 min, and chain extension at 72°C for 2 min. Amplified products were extracted from a 1.5% TAE agarose gel with a Wizard Miniprep DNA Purification Kit (Promega Corporation, Madison, Wisconsin, USA), according to manufacturer instructions. Sequences were cloned using the Invitrogen TOPO Top 10F cloning kit, according to manufacturer instructions (Carlsbad, California, USA). Plasmids were lysed in TE Buffer at 96°C for 10 min and screening for the correct insert size per clone was done by using 0.7% TBE electrophoresis gels and ethidium bromide staining.

### Sanger sequencing and analysis

Cloned plasmids containing correctly-sized amplification products were sequenced with the plasmid-specific primers M13(-20) (5′-GTAAAACGACGGCCAGT-3′) and M13(−24) (5′-AACAGCTATGACCATG-3′) and with the 907R 16S rRNA internal primer (5-CCG TCA ATT CMT TTR AGT TT-3′), using capillary sequencers (Sanger sequencing) at the High-Throughput Genomics Unit at the University of Washington in Seattle (USA).

A total of 924 sequences were successfully assembled using Contig Express from Vector NTI Advance 10.3.0 (Invitrogen Corp., USA), then sequences were checked for chimera using Bellerophon (Huber et al., 2004) in mothur (Schloss et al., 2009). Potential chimeric sequences were manually checked by conducting a BLAST search (http://www.ncbi.nlm.nih.gov/) and 9.3% (86 sequences) of the sequences were removed. The final dataset was submitted to the Ribosomal Database Project (RDP) Classifier to determine taxonomic affiliations for the 16S rRNA genes (Wang et al., 2007). Sequences with <80% similarity to their closest relative(s) were considered unidentified. Sequences were clustered using RDP complete linkage program (Cole et al., 2007, 2009) at a 96% cutoff to define OTUs and to calculate rarefaction curves. Shannon diversity scores (H′) were calculated using the RDP Shannon Index and Chao1 estimator, including evenness (E) (Shannon, 1948; Chao, 1984).

### 454 pyrosequencing and analysis

For massively parallel bacterial tag-encoded FLX amplicon pyrosequencing, DNA extractions from proximal sites with similar geochemistry were combined (Table 1) for Sharon Springs (i.e., 5 μl of each DNA extraction from the cistern and drinking spring), and all three sample sites in Sulphur Springs (i.e., 2 μl of each DNA extraction plus 4 μl of a mixture of the extractions to equal 10 μl total). For all the other samples, 10 μl of extracted DNA from each sample were used for pyrosequencing. The V1-V3 region of the 16S rRNA gene was amplified and all sequencing was done by the Research and Testing Laboratories (Lubbock, Texas, USA), using previously described methods (Dowd et al., 2008a,b).

Following pyrosequencing, all failed reads, low quality score sequences (<20), and non-bacterial ribosomal sequences were removed. The remaining data set consisted of 79,681 pyrosequences that were checked for chimera using Bellerophon (Huber et al., 2004). Approximately 26% of the sequences were potentially chimeric and removed. Taxonomy was assigned using the RDP Classifier (Wang et al., 2007). Sequences with <80% similarity were classified as unidentified bacteria. When an OTU had the majority of its sequences equal to or greater than 94% sequence similarity to a closest relative belonging to a known genus, we classified that OTU by the genus name. However, if the majority of sequences in an OTU had less than 94% sequence similarity to a known genus, we classified the OTU as genus “-like” to clarify that the genus is similar, but likely unknown. Pyrosequences were clustered at a 96% cutoff using RDP to form OTUs that were used to calculate rarefaction curves. Diversity indices were also calculated from RDP, including H′ and E. OTUs shared across sites were assessed to evaluate beta-diversity.

### Statistical analyses

Relationships between environmental factors (e.g., pH, sulfide, etc.) and normalized abundances of major taxonomic groups were analyzed by stepwise canonical correspondence analysis (CCA) using the PAST software (version 2.14) (Hammer et al., 2001). Variables were sequentially removed to maximize correlations between principal axes and linear combinations of environmental variables (Legendre and Legendre, 1998). Significance from permutation tests required a *P*-value <0.05.

### NCBI sequence accession numbers

Near full-length 16S rRNA gene sequences were deposited in GenBank under the accession numbers JX520968—JX521805. Raw pyrosequence reads were submitted to the NCBI Sequence Read Archive under study number SRA080388.

## Results

### Spring geochemistry

Sulfide concentrations at the springs were relatively low compared to other terrestrial sulfidic springs reported in the literature (e.g., Elshahed et al., 2003; Perreault et al., 2007) (Table 1). Spring water temperature ranged from 7.5°C at Richfield Springs to 45.3°C at SCHS. pH ranged from 7.07 at Richfield Springs to 9.75 at TV2, and conductivity ranged from 647 μS/cm at Palmetto Springs to 32,800 μS/cm at TV2. Sharon and Richfield Springs had comparable geochemistries, likely because they are sourced from the same aquifer. Sulphur Springs, OK, had similar conditions to Sharon and Richfield Springs. The pH, conductivity, and sulfide concentrations differed for TV1 and TV2 springs, despite being closest to each other than any other springs sampled. They did have similar temperatures and dissolved oxygen content (Table 1). The dissolved oxygen content was generally low for all of the springs except for Vendome well, likely because the well water is aerated while being sprayed into the air.

### Taxonomy of shared and unique 16S rRNA genes

**Figure 2:**
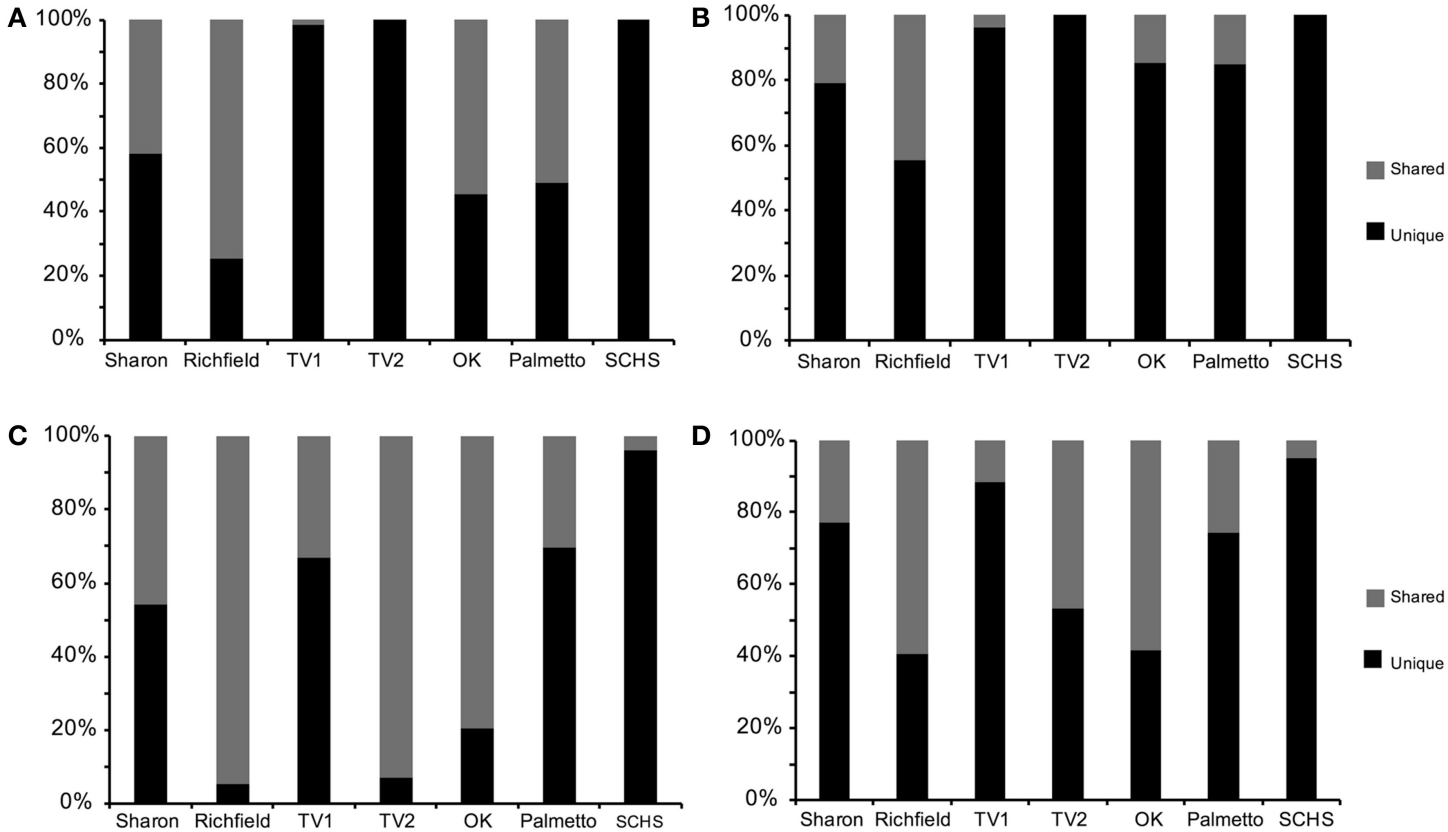
**Percentage of shared vs. unique (A) near full-length 16S rRNA gene sequences from Sanger sequencing and (B) operational taxonomic units (OTUs) from Sanger sequences, and (C) pyrosequences of 16S rRNA genes and (D) OTUs from pyrosequences (Sharon, Sharon Springs; Richfield, Richfield Springs; TV1, Tully Valley 1; TV2, Tully Valley 2; OK, Sulphur Springs; Palmetto, Palmetto Springs; SCHS, South Canyon Hot Springs)**.

Comparable taxonomic results were obtained from the 838 full-length 16S rRNA gene sequences (Supplemental Table 1) and 58,912 pyrosequences (Supplemental Table 2). Taxonomic representation among OTUs was also similar between the Sanger and pyrosequencing datasets (Supplemental Table 3), although pyrosequencing resulted in more deeply sampling the taxonomic diversity compared to Sanger sequencing (Figure 2). From the Sanger sequences, 251 OTUs represented five proteobacterial classes, 11 other major phyla, and two candidate divisions, with 65.9% (552 sequences) being identifiable to the genus level (Supplemental Table 4A). From the pyrosequences, 3481 OTUs also represented five proteobacterial classes, but 31 other major phyla and five candidate divisions were identified, with 26.8% (15,821) of the pyrosequences being classified to the genus level (Supplemental Table 4B). Diversity indicators (e.g., H′ and E) varied for the springs due to differences in the overall diversity detected by the two sequencing methods (Table 2). The numbers of 16S rRNA genes retrieved from Sanger sequencing and pyrosequencing for each dataset were used to indicate the relative abundances of particular bacterial groups at a spring. Unique sequences were considered to be those that were retrieved from only one spring and did not form an OTU with sequences from other springs. Shared OTUs were comprised of sequences from more than one spring, thereby suggesting shared communities but not necessarily a contemporary physical connection between the springs.

**Table 2:** **Diversity indices of 16S rRNA pyrosequences**.

**Sample site**	**Beta diversity^a^**							**Distance**	**N^b^**	**Chao1**	**H'**	**E**
	**S**	**R**	**TV1**	**TV2**	**OK**	**P**	**SCHS**					
Sharon Springs (S)	**0/**0	**0.62295/**0.59322	**0.73437/**0.67213	**0.76290/**0.73333	**0.70000/**0.61290	**0.89286/**0.81818	**0.92308/**0.83607	0.04	9185	386 (350–448)	3.4	0.6
Richfield Springs (R)		**0/**0	**0.84466/**0.80952	**0.88372/**0.85366	**0.75000/**0.67442	**0.94444/**0.88889	**0.97778/**0.95238	0.04	8272	1632 (1538–1752)	5.7	0.8
Tully Valley 1 (TV1)			**0/**0	**0.64486/**0.58140	**0.94059/**0.86667	**0.97849/**1	**0.94595/**0.90909	0.04	22968	1619 (1519–1749)	4.6	0.7
Tully Valley 2 (TV2)				**0/**0	**0.90475**/0.86364	**0.97386/**0.94595	**0.93617/**0.86047	0.04	8808	356 (310–436)	2.4	0.4
Vendrome Well, Sulphur Springs (OK)					**0/**0	**0.85714/**0.74359	**0.95455/**0.91111	0.04	2193	150 (127–197)	2.6	0.6
Palmetto Springs (P)						**0/**0	**0.82500/**0.78947	0.04	1163	175 (140–244)	2.8	0.6
South Canyon Hot Spring (SCHS)							**0/**0	0.04	6323	1017 (888–1199)	4.2	0.7

a ***Beta Diversity of full community/** Beta Diversity of only putative sulfide-oxidizing bacteria*.

b Number of pyrosequences.

Among the pyrosequences, 12% of the OTUs (427 OTUs, comprised of 30,625 pyrosequences) consisted of reads from more than one spring. Consequently, much of the taxonomic diversity was not shared between the springs. The number of shared sequences per spring ranged from 3.6% (SCHS) to as high as 94.7% (Richfield) (Figure 2C). Only 44 OTUs (9,123 sequences, or 15% of the total number of sequences in the dataset) were shared among more than two springs in large numbers (i.e., >1% of sequences). Sharon, TV1, Palmetto, and SCHS had more unique than shared OTUs (Figure 2D). Only one OTU, belonging to the *Thiofaba* spp. (50–100% sequence similarity) (*Gammaproteobacteria*), formed from pyrosequences retrieved from five springs (Sharon, Richfield, TV1, TV2, and Sulphur Springs) (Supplemental Table 4B). Moreover, despite some OTUs having shared sequences from multiple springs, there was variability in the taxonomic affinities for sequences comprising the specific OTUs. For instance, sequences from the TV1 and TV2 springs that formed OTUs closely related to the genus *Sulfurimonas* (*Epsilonproteobacteria*) had the highest sequence similarity to the genus, with 70% of the sequences being 94% or greater sequence similarity to the genus. In contrast, sequences within the OTU from Sharon, Sulphur Springs, and SCHS had <94% sequence similarity to the genus *Sulfurimonas.*

Most of the shared OTUs, defined as OTUs comprised of sequences from more than one spring, were retrieved from only two sites and were comprised of abundant pyrosequences that belonged to just a few taxonomic groups, such as the putative sulfur-oxidizing *Epsilonproteobacteria* and *Gammaproteobacteria* (Supplemental Table 4B). OTUs comprised of high numbers of shared pyrosequences were found for Sharon and Richfield springs, NY, and Sulphur Springs, OK. Richfield Springs shared 51.5% of its epsilonproteobacterial population with Sharon and Sulphur Springs, whereas Sulphur Springs shared 52% of its gammaproteobacterial population with Sharon and Richfield Springs. Deltaproteobacterial pyrosequences were only shared among the New York springs, with Sharon Springs sharing 53% of its deltaproteobacterial population with Richfield Springs (Richfield Springs shared 79% of its deltaproteobacterial population with Sharon Springs) and TV1 shared 41% of its deltaproteobacterial population with TV2 (TV2 shared 93% of its deltaproteobacterial population with TV1). Only two deltaproteobacterial OTUs were shared between TV springs and Sharon and Richfield springs (Supplemental Table 4B).

### Geochemical and geographic distribution of OTUs

**Figure 3:**
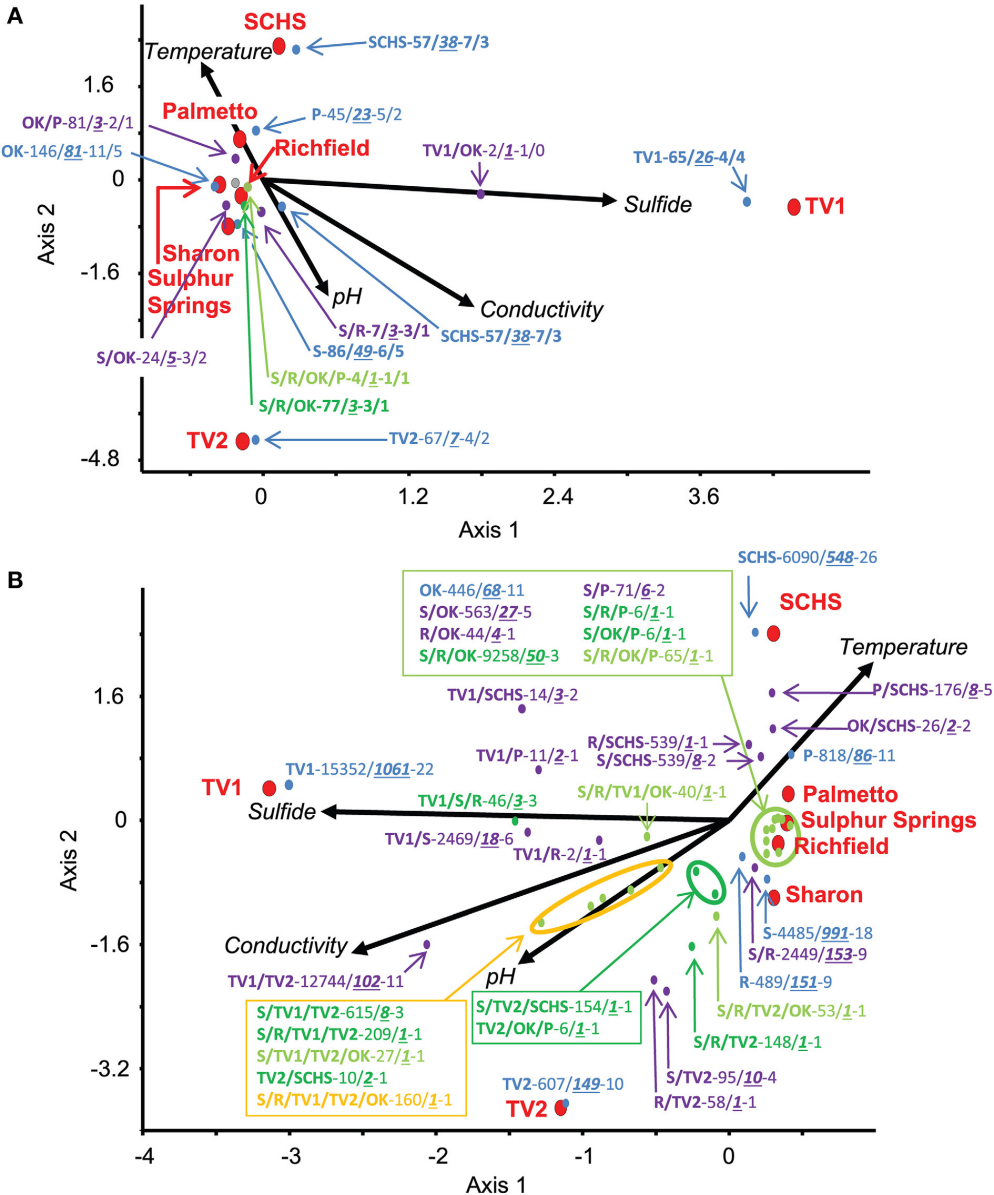
**Canonical Correspondence Analysis (CCA) for the study springs, abbreviated as Sharon (S), Sharon Springs; Richfield (R), Richfield Springs; TV1, Tully Valley 1; TV2, Tully Valley 2; OK, Sulphur Springs; Palmetto (P), Palmetto Springs; SCHS, South Canyon Hot Springs. (A)** CCA of OTUs of near-full length 16S rRNA gene sequences produced from Sanger sequencing (*P*-value = 0.02). Red dots represent the sampled springs. Colored dots represent OTUs comprised of different numbers of springs that are labeled in the following order: Site(s)—No. of 16S rRNA sequences/No. of OTUs—No. of major phyla (including Proteobacteria)/No. of proteobacterial classes. **(B)** CCA of OTUs from 16S rRNA gene pyrosequences (*P*-value = 0.01). Red dots represent sampled springs. Colored dots correspond to pyrosequence OTUs from springs that are labeled as: Site(s) - No. of 16S rRNA sequences/No. of OTUs—No. of major phyla (including Proteobacteria). For both CCAs, arrows represent environmental gradients.

Relationships among the geographic distributions of taxonomic groups to spring geochemistry were verified by CCA from the Sanger sequences and pyrosequences separately using the geochemical variables of pH, conductivity, sulfide, and temperature. For the near full-length Sanger sequences (Figure 3A), CCA axis 1 correlated to sulfide (38% of the variance) and CCA axis 2 correlated to temperature and pH (33% of the variance) (*P*-value = 0.02 following 100 Monte Carlo permutations). For the pyrosequence data (Figure 3B), 73% of the variance could be described by two axes of the final CCA (*P*-value = 0.009 following 100 Monte Carlo permutations). Similar to the results from the Sanger sequence analyses, CCA axis 1 for the pyrosequence results correlated to sulfide (37% of the variance), and CCA axis 2 correlated to temperature and pH (36% of the variance). Other variables, such as dissolved oxygen concentration, probable source(s) of the springs (i.e., sandstone vs. carbonate, etc.), and spring environment (i.e., concrete vs. “natural”), were evaluated and found not to be statistically significant in any of the analyses.

**Figure 4:**
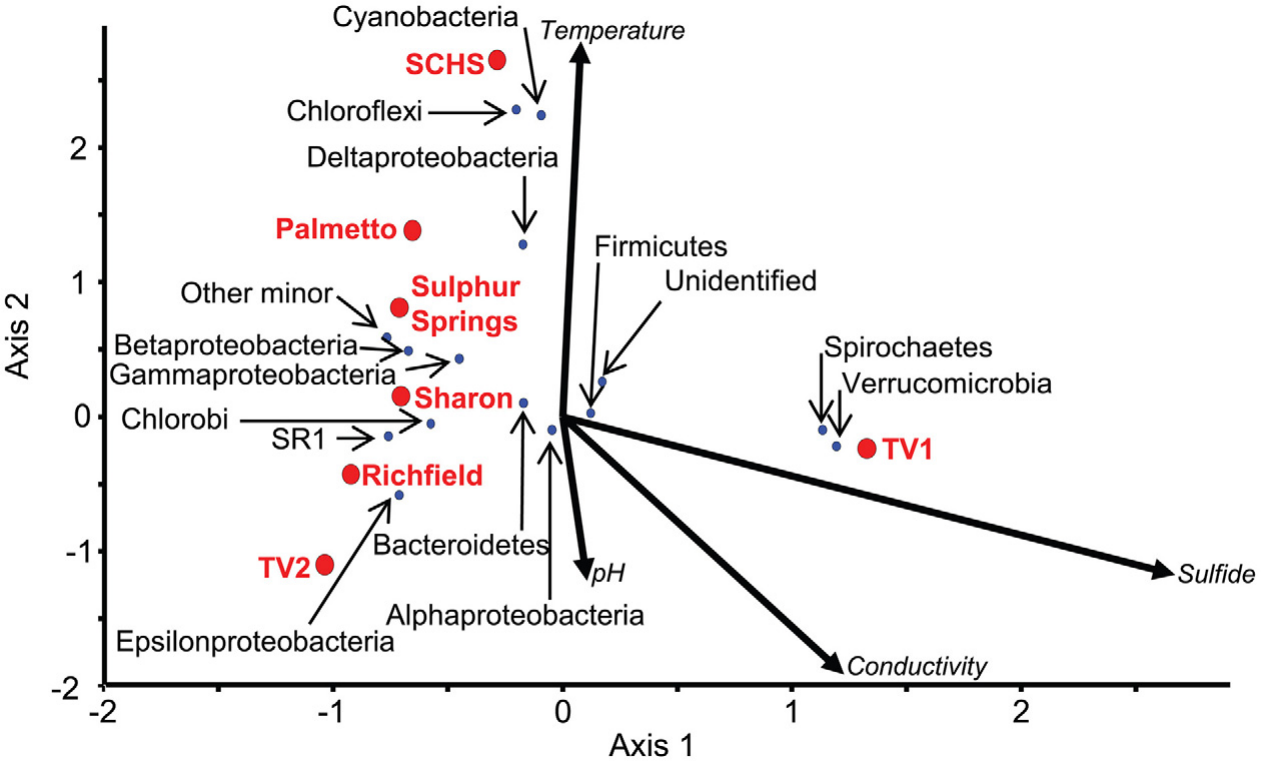
**CCA of 16S rRNA pyrosequences summarized by major taxonomic group (*P*-value = 0.04)**. Red dots represent the sampled springs. Blue dots represent OTUs defined by 16S rRNA gene pyrosequences for major taxonomic groups from the springs. Arrows represent environmental gradients.

The ordination of the overall diversity from the pyrosequence data for each of the sites against geochemistry correlated to the ordination of major taxonomic groups at those sites (Figure 4; Supplemental Table 3). For instance, the communities from Sharon, Richfield, Sulphur, and Palmetto springs negatively correlated to the vector directions for sulfide concentration, conductivity, and pH, although Palmetto Spring communities positively correlated to the vector attributed to temperature. For each of these springs, *Epsilonproteobacteria* and *Gammaproteobacteria* dominated the communities and these groups were closely ordinated to the spring sites in the CCA analysis (Figure 4). The community from TV1 positively correlated with sulfide and conductivity, but the TV2 community positively correlated with pH and conductivity and negatively to temperature. Both Verrucomicrobia and Spirochaetes, which dominated the TV1 microbial community (Supplemental Table 3), were ordinated with the overall TV1 community position and the sulfide vector (Figure 4). SCHS had the highest temperature of all sites (Table 1), and the overall SCHS community positively correlated with the temperature vector, and also ordinated with Cyanobacteria, Chloroflexi, and *Deltaproteobacteria* (Figure 4).

Examination of the genus-level diversity indicated that genera comprising major OTU groups were much more diverse than suggested from the analyses resulting in Figure 4. Genera specific to the springs in which they were found correlated to the geochemical conditions at those springs (Figures 3A,B). For instance, sequences related to *Geobacter* spp. (77–100% sequence similarity) were the dominant *Deltaproteobacteria* at Sharon Springs, but sequences related to *Geopsychrobacter*-like (1–62% sequence similarity) and *Desulfuromusa* (51–100% sequence similarity) spp. were the dominant *Deltaproteobacteria* at TV1 (Supplemental Table 4B). *Geobacter* spp. correlated to the low sulfide conditions at Sharon Springs, but *Geopsychrobacter*-like and *Desulfuromusa* spp. correlated to the high sulfide concentrations associated with TV1 (Figure 3B). Sequences shared between the sites plotted on the CCA graphs halfway between the shared sites and correlated to (i.e., or were indicative of) the geochemical parameter that would have governed their dominance at the site. Shared sequences related to *Thiothrix* spp. (43–100% sequence similarity) from Sulphur and Palmetto springs correlated to high temperature, whereas sequences related to *Thiothrix* spp. shared between Sharon and Richfield springs correlated to lower temperatures and sulfide conditions (Figure 3B; Supplemental Table 4B). TV1 and TV2 shared sequences related to *Alterococcus*-like (10–85% sequence similarity) spp. (Verrucomicrobia) that correlated to high sulfide and conductivity, even though higher sulfide concentrations were only found in TV1. However, the distribution of sequences comprising this OTU was uneven, as most of the sequences were from TV1 (Supplemental Table 4B).

Compared to the statistical support for geochemical constraints on bacterial community distribution, geographic distance did not appear to be an important regulator of microbial community composition at any of the spring locations based on bacterial presence/absence data. Sharon and Richfield Springs, which were 30 km apart and likely affected by the same hydrogeological aquifer conditions, had similar OTUs in common with each other. Also, TV1 and TV2, which were a few meters apart from one another, also shared OTUs in common with one another. But, TV1 had more bacterial OTUs in common with Sharon Springs (125 km distance) than TV1 did with Richfield Springs (95 km distance). Moreover, Sharon and Richfield springs shared more OTUs in common with Sulphur Springs (~2100 km) than these springs did with TV1 and TV2 collectively. SCHS shared more of its putative sulfur-oxidizing bacterial OTUs with Sharon Springs (2753 km away) and Palmetto Spring (1426 km away) then it did with Sulphur Springs (1083 km away) (Supplemental Table 4B).

## Discussion

Understanding the variables that govern the geographic distribution of bacteria in the environment is a complicated task, particularly given that many variables likely constrain microbes to specific habitats (e.g., Green et al., 2008). This study examined the distribution of 16S rRNA genes across geochemically diverse, terrestrial sulfidic springs to identify common microbial communities to test the hypothesis that a core microbiome for sulfidic springs would be linked to similar geochemical conditions and not geographic distance. In general, the results from our investigation support the hypothesis.

### Geochemical controls on the distribution of bacterial groups

The most numerically dominant bacteria in each spring were associated with the classes *Epsilonproteobacteria* and *Gammaproteobacteria*, these groups also shared more sequences in common with other geochemically similar sites. But, there were also high numbers of OTUs retrieved that were unique to the springs within these taxonomic groups (Figure 2; Supplemental Tables 4A,B). Other studies examining microbial diversity at varying scales in different environments like deep sea vents (Opatkiewicz et al., 2009), thermal springs (Lau et al., 2009), sulfidic springs (Youssef et al., 2010), and soils (Roesch et al., 2007) have similar results. Some of the groups that were unique to the springs in our study also comprised the major groups found at each of the springs, which suggested that these bacteria are adapted specifically to the conditions of their respective spring (Supplemental Tables 3, 4B). It is not possible to determine if the 16S rRNA sequences of the less abundant groups represent well adapted lineages that serve as a microbial seed bank or bacteria that are transient.

**Figure 5:**
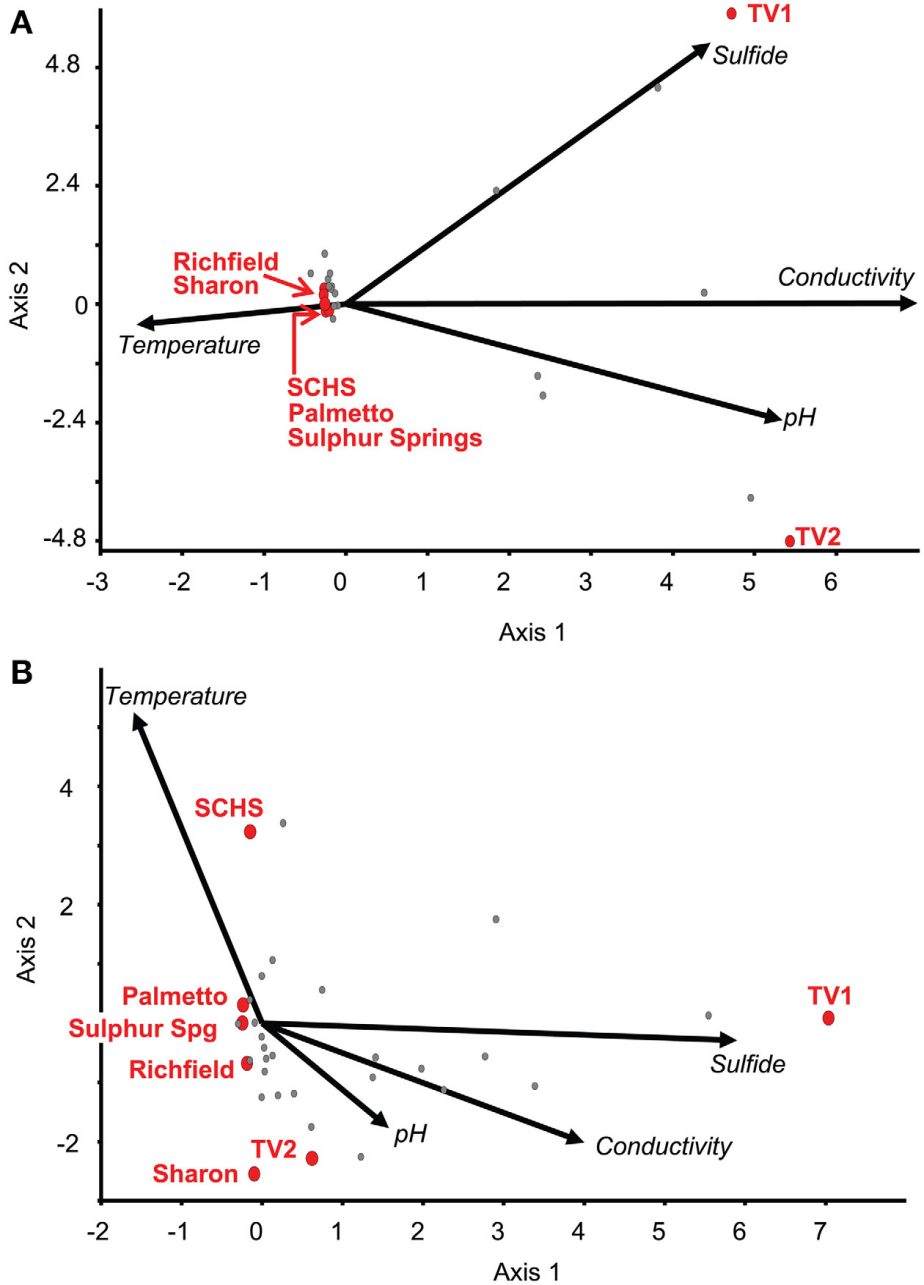
**CCAs of 16S rRNA pyrosequence OTUs belonging to (A) *Epsilonproteobacteria* and (B) *Gammaproteobacteria***. Red dots represent the ordination of the sampled spring diversity. Gray dots represent OTUs defined by 16S rRNA gene pyrosequences for each class from the springs. Arrows represent environmental gradient.

The geochemical conditions associated with many terrestrial sulfidic springs are suitable to both *Epsilonproteobacteria* and *Gammaproteobacteria*, though these two groups clearly occupy different niches (Macalady et al., 2008; Headd and Engel, 2013). At the genus-level, the distribution of specific genera within the *Epsilonproteobacteria* spanned the entire measured geochemistry of the springs examined in our study (Figure 5A) and strongly correlated to the geochemistry at sites of opposing geochemistries. Similar results were observed among the *Gammaproteobacteria* (Figure 5B), specifically for the genera *Thiofaba* (50–100% sequence similarity) and *Thiothrix* (43–100% sequence similarity). The distribution of *Epsilonproteobacteria* in sulfidic springs has previously been associated with carbonate-hosted systems (Porter and Engel, 2008). In general, *Epsilonproteobacteria* in our study were relatively common bacterial community members in all of the springs, except Palmetto (0.77% of total community based on pyrosequencing data) and SCHS (1.18% of total community). Also, the majority of the pyrosequences related to *Epsilonproteobacteria* were shared among the carbonate-hosted springs, as opposed to being unique to the individual springs, with the exception of SCHS (Supplemental Table 4B). Palmetto Spring is a non-carbonate system and SCHS, which may also not be a carbonate-hosted system, has water temperatures at the highest end of what has been previously reported for the presence of terrestrial epsilonproteobacterial lineages (Porter and Engel, 2008; Rossmassler et al., 2012).

The distribution and occurrence of *Epsilonproteobacteria* as a class correlated to low sulfide concentrations in the springs (Figure 5). This finding contrasts results from deep-sea vents where *Epsilonproteobacteria* thrive at much higher sulfide concentrations than the springs we examined (Akerman et al., 2013). Terrestrial epsilonproteobacterial lineages are distantly related to marine lineages and appear to have adapted to terrestrial environments with lower temperatures and sulfide concentrations (Engel et al., 2004; Campbell et al., 2006; Hubert et al., 2012). *Epsilonproteobacteria* prefer lower oxygen environments (Engel et al., 2004; Macalady et al., 2008; Grote et al., 2012), some can reduce nitrate, and some lineages can oxidize hydrogen as an electron donor (Campbell et al., 2006; Yamamoto and Takai, 2011) but this requires a low oxygen environment. These metabolisms could restrict the colonization of *Epsilonproteobacteria* in terrestrial surface environments open to atmospheric levels of oxygen, even if sulfide concentrations are sufficient for metabolism.

*Gammaproteobacteri*a were identified across a larger geographic area than the epsilonproteobacterial groups in our study. It is unclear if this is a sampling issue or if it is due to the adaptability of sulfide-oxidizing *Gammaproteobacteria*, mostly related to *Thiofaba* and *Thiothrix* spp. *Thiofaba* spp. are aerobic sulfide-oxidizing bacteria commonly found in hot springs (Mori and Suzuki, 2008; Akimov et al., 2013). *Thiofaba* spp. were most prominent in SCHS, which had the highest temperature of the springs we examined. *Thiothrix* spp. are also common to terrestrial sulfidic springs (Engel et al., 2004; Rudolph et al., 2004; Macalady et al., 2008; Chaudhary et al., 2009; Chernousova et al., 2009; Headd and Engel, 2013) and tend to dominate oxygenated waters with lower sulfide concentrations (Engel et al., 2004; Macalady et al., 2008; Headd and Engel, 2013). With the exception of TV2 (Table 1) which had few sequences affiliated with *Thiothrix* spp. (Supplemental Table 4B), oxygenated, low sulfide waters were found at all of the springs we examined, particularly the outflow channels that can be meters in length. In contrast, the areas around an orifice or at depth in microbial mats are generally anaerobic, and although sulfide concentrations may be high enough, the lack of oxygen would not support the metabolism of *Thiothrix* spp. (Headd and Engel, 2013). However, it is possible that the widespread occurrence of *Thiothrix* spp. in terrestrial sulfidic springs may be due to a its potential to colonize a geochemically wider range in the environment (and ease of sample collection) relative to other sulfide-oxidizing bacteria that are restricted to more narrow geochemical ranges.

Although several other bacterial groups (e.g., Bacteroidetes) were prevalent members of the communities at each spring, in addition to *Epsilonproteobacteria* and *Gammaproteobacteria* (Supplemental Table 3), the percentage of shared OTUs among other bacterial groups was low (<1.0%). In five of the seven springs, Bacteroidetes comprised one of the top three most abundant major taxonomic groups, but shared OTUs were comprised of relatively few 16S rRNA gene sequences (Supplemental Tables 3, 4A,B). Bacteroidetes are commonly associated with sediments/soils in terrestrial environments (e.g., Roesch et al., 2007) and gut microbiomes of wildlife (Lamendella et al., 2007), so we interpret their presence in the springs as being due to sourcing from nearby sediment or soil communities surrounding the mats that were sampled or possibly from wildlife visiting the springs.

Temperature was also an important control on microbial community distribution. SCHS had the highest temperature and proportionately shared the least of its microbial population with the other springs studied. Temperature was a defining feature for SCHS, and although it is at the low end of the temperature spectrum for a thermal spring, it is likely that most of the OTUs unique to SCHS are adapted to the higher temperature and are not well suited for lower temperature springs examined. Therefore, less of the SCHS microbial community was shared with the other springs because the dominant microbial groups found in SCHS closely resembled the higher temperature microbial groups commonly found in other thermal sulfidic springs (Skirnisdottir et al., 2000; Lau et al., 2009; Kubo et al., 2011; Akimov et al., 2013).

Additional variables may also impact our interpretation of controls on the distribution of microbial populations, including geochemical properties that we did not sample and even the way samples were obtained. Collecting several grams of material from each spring, comprised of an infinitely-sized microbial community within undefined habitat dimensions, may not be equally representative of a community at a specific spring. Within a habitat, microbial communities are unevenly arranged, and some samples are likely to contain more or less amounts of the neighboring microbial communities that may or may not have an equivalent presence in a sample from a different spring, even if geochemical conditions are the same. Because pyrosequencing has the potential to evaluate diversity more deeply compared to clone-based Sanger sequencing, it is possible that the majority of pyrosequences in the “tail” of abundance data, often regarded as “rare biosphere” (Sogin et al., 2006; Galand et al., 2009) when obtained from single samples, could be members of neighboring microbial communities that have not been properly sampled (Youssef et al., 2010).

### Sulfidic spring core microbiome

From comparing the distributions of epsilonproteobacterial and gammaproteobacterial groups, a core sulfidic spring microbiome becomes apparent. Populations of relatively abundant *Hydrogenimonas*-like (8–75% sequence similarity) or *Sulfurovum*-like (3–99% sequence similarity) species from the *Epsilonproteobacteria* occurred in springs (Sharon, Richfield, and Sulphur Springs) with relatively abundant *Thiothrix* and *Thiofaba* spp. (*Gammaproteobacteria*) populations. But, populations related to *Arcobacte*r-like (22–100% sequence similarity) and *Sulfurimonas* spp. (20–100% sequence similarity) (*Epsilonproteobacteria*) were more abundant where populations of *Thiofaba* and *Thiothrix* spp. had relatively low relative abundances (specifically at TV1 and TV2). Despite the presence of some genera in many, if not all, of the spring communities, such as *Wolinella*-like spp. (2–100% sequence similarity) (*Epsilonproteobacteria*), OTUs affiliated to these genera did not form with sequences from more than one or two of the springs. This implies that these OTUs were not taxonomically similar to each other and further indicates that much of the retrieved spring community composition is specific to the spring conditions in which the groups are found.

In contrast, OTUs that formed with sequences from three or more spring communities are essentially the centroid of a community that represents a core microbiome for sulfidic springs at their respective geochemistries. This core includes eight OTUs, one affiliated with *Epsilonproteobacteria* and seven with *Gammaproteobacteria*. The significance of these overlapping communities is unclear, but may suggest that the co-occurrence of specific epsilonproteobacterial and gammaproteobacterial groups is regulated, in part, by geochemistry at each of the habitats and that springs with similar geochemistry have a common microbiome, while those with dissimilar geochemistries do not share taxonomic groups. To our knowledge, no one has proposed a sulfidic spring microbiome, although Probst et al. (2013) defined a core microbiome for a biofilm dominated by the SM1 euryarchaeon collected from a sulfidic spring. The authors of this study suggested that the SM1 euryarchaeon may be regulating the proteobacterial groups in the biofilm (Probst et al., 2013). We did not examine Archaea in this study, but it is possible that biology, in addition to geochemistry, may regulate the microbial communities in the springs we examined.

### Long-term geologic processes and present-day biogeographic patterns

Recent studies have provided evidence that global microbial biogeography is not determined by contemporary dispersal, but by long-term geologic processes ranging from sediment deposition to plate tectonics (Johnson et al., 2007; Bahl et al., 2011; Strunecky et al., 2012). Burial does not mean extinction for bacteria (Kieft et al., 1998; Johnson et al., 2007) and non-sporulating bacteria have been shown to enter states of dormancy that can ensure their survival for long periods of time in environments that do not favor their growth (Mulyukin et al., 2003; Whittington et al., 2004). It is unclear how long bacteria can remain viable in the subsurface, but it has been proposed that some can remain viable for millions of years (e.g., Vreeland et al., 2000; Johnson et al., 2007). As Kieft et al. (1998) noted, the original cells of a surface community buried in the subsurface may not survive, but they may need only replicate a few times to ensure survival of the lineage. Microorganisms buried in the subsurface can be brought back to the surface via springs and establish or join microbial communities where and when the geochemical conditions are favorable (Tin et al., 2011). Other microbial dispersal methods (e.g., wind, insects, etc.) have also likely contributed to the distribution of microbes at each of the springs, and could be used to explain how similar taxonomic groups could be found in geographically separated springs. Thus, through cycles of burial and re-surfacing, as well as indirect distribution due to other processes, terrestrial surface microbial communities could be dispersed over vast areas of a continent (and multiple continents via the breakup of supercontinents) through geologic time.

Although we did not find any correlations between geographic distance and the distribution of microbial groups among the springs examined, there were still peculiar distribution patterns among large numbers of 16S rRNA sequences that were shared between the springs. For example, Palmetto Springs shared 10 OTUs (592 pyrosequences) with Sulphur Springs and 8 OTUs (173 pyrosequences) with SCHS. But, SCHS and Sulphur Springs only shared 2 OTUs (26 pyrosequences) with one another (Supplemental Table 4B). None of these springs have an obvious contemporary geographic or hydrological connection. One possible explanation for the microbial distribution pattern for Palmetto, SCHS, and Sulphur springs, then, could be that terrestrial microbial communities inhabiting paleo-terrestrial sulfidic environments in the Rocky (SCHS) and Arbuckle (Sulphur Springs) mountains were transported along with eroded sediments by paleo-fluvial processes and ultimately deposited in the Wilcox Group sediments (the stratigraphy at Palmetto Springs). The Wilcox Group sediments are derived from the central and southern Rocky Mountains, Oklahoma and northern and western Texas (Mackey et al., 2012). There is no reason to believe that terrestrial sulfidic springs are a modern occurrence and it is probable that contemporary microbial groups inhabiting sulfidic springs evolved from groups inhabiting paleo-sulfidic springs or similar terrestrial sulfidic environments. The microbial communities transported away from these ancient environments could have been buried in the subsurface and entered a dormant stage to ensure their survival. While many of the microbial lineages may have perished or have yet to re-surface, some would have re-surfaced in geochemically favorable environments, such as sulfidic springs, and established surface communities. This cycle could continue (multiple times, perhaps) to produce their present biogeographical distribution. It is unclear how long Palmetto Springs has been flowing, but it is important to note that bacterial lineages should be presumed to be older than the age of groundwaters they inhabit (Balkwill et al., 1998). Therefore, the source waters for all three of these springs could be much younger than the bacteria inhabiting the waters.

Sulphur Springs and the New York Springs shared a large number of OTUs, but we were unable to establish a clear paleo-connection between these springs. This is due to a lack of available paleogeographic information in the literature. The New York springs were too far west to be included in the Greenville orogeny (Berdan, 1950), but it is possible that paleodrainage systems carried sediments from the New York area toward the south and west, possibly as far west as Oklahoma. But, the resolution needed to establish a connection between Sulphur Springs and the New York springs is not currently available.

In conclusion, we propose that the terrestrial springs serve as a long-term microbial transport mechanism that links the subsurface and surface environments through geologic time. In this study, the springs examined were all sulfidic and when bacteria with sulfur-dependent metabolisms emanate at the springs (i.e., with suitable geochemical conditions), they proliferate, while those bacteria that are not adapted to the geochemical conditions do not proliferate and thus, are only recovered in low numbers. These bacteria may be transported by surface processes to other environments or may be quickly buried in the sediments within and surrounding the springs. The source areas for these springs likely include sediments and microbial communities from a wide range of environments (e.g., forests, rivers, lakes, soils, etc.) that existed over the span of the millions of years it took to deposit the sediments and microbes in the sources of the springs we examined. This scenario suggests it might be possible to utilize contemporary terrestrial spring microbial communities to obtain paleogeographic and paleoecological information, and to evaluate how congruent biogeographic distribution patterns of microbial communities may change in the future with climatic and anthropogenic impacts. This scenario could help to explain how core microbiomes are dispersed, transported, and become established in sulfidic springs, as well as why closely related taxa speciate and become taxonomically distinct from each other due to specific geochemical conditions at each location through time.

## Author contributions

Brendan Headd designed and conducted the research, conducted data analysis and interpretation and drafted and reviewed the manuscript. Annette S. Engel designed the research, conducted data analysis and interpretation, and reviewed the manuscript. Both Brendan Headd and Annette S. Engel agree to be accountable for all aspects of the work and to ensure that questions related to the accuracy or integrity of any part of the work are appropriately investigated and resolved.

### Conflict of interest statement

The authors declare that the research was conducted in the absence of any commercial or financial relationships that could be construed as a potential conflict of interest.
