# Supplementary material for: Biogeographic congruency among bacterial communities from terrestrial sulfidic springs
Source: Front Microbiol. 2014 Sep 8;5:473. doi: 10.3389/fmicb.2014.00473 (PMC4157610; doi:10.3389/fmicb.2014.00473)
Supplement: Supplementary file 1 [file Table1.DOCX]

Supplemental Table 1. Numbers of sequences (and number of OTUs) for each of the major taxonomic groups from near full-length 16S rRNA genes from Sanger sequencing.

| Major Taxonomic Group | Sharon | Richfield | TV1 | TV2 | Sulphur | Palmetto | SCHS |
| --- | --- | --- | --- | --- | --- | --- | --- |
| *Alphaproteobacteria* | 4 (4) | 0 | 1 (1) | 0 | 11 (10) | 0 | 0 |
| *Betaproteobacteria* | 10 (5) | 0 | 0 | 0 | 8 (2) | 5 (5) | 4 (3) |
| *Deltaproteobacteria* | 9 (7) | 11 (1) | 3 (3) | 0 | 4 (4) | 0 | 4 (4) |
| *Epsilonproteobacteria* | 22 (4) | 18 (2) | 5 (1) | 46 (2) | 37 (6) | 0 | 0 |
| *Gammaproteobacteria* | 36 (10) | 26 (5) | 9 (6) | 2 (2) | 146 (16) | 67 (11) | 10 (1) |
| Acidobacteria | 0 | 0 | 0 | 0 | 2 (2) | 0 | 0 |
| Bacteroidetes | 49 (21) | 22 (3) | 11 (6) | 0 | 42 (23) | 7 (4) | 6 (6) |
| Chlorobi | 0 | 0 | 0 | 5 (1) | 20 (2) | 0 | 3 (3) |
| Chloroflexi | 2 (1) | 1 (1) | 0 | 0 | 5 (5) | 0 | 15 (10) |
| Cyanobacteria | 0 | 0 | 0 | 2 (1) | 22 (10) | 8 (3) | 6 (2) |
| Eukaryota | 0 | 0 | 1 (1) | 0 | 1 (1) | 0 | 0 |
| Firmicutes | 0 | 0 | 0 | 0 | 1 (1) | 0 | 1 (1) |
| Nitrospira | 0 | 0 | 0 | 0 | 1 (1) | 0 | 0 |
| Planctomycetes | 1 (1) | 0 | 0 | 0 | 0 | 0 | 0 |
| Spirochaetes | 0 | 0 | 6 | 0 | 0 | 0 | 0 |
| Candidate Division OD1 | 1 (1) | 0 | 0 | 0 | 1 (1) | 0 | 0 |
| Candidate Division SR1 | 4 (2) | 9 (3) | 0 | 0 | 4 (2) | 2 (1) | 0 |
| Verrucomicorbia | 0 | 0 | 27 (3) | 0 | 0 | 1 (1) | 0 |
| Unidentified | 10 (6) | 0 | 3 (2) | 12 (1) | 16 (9) | 2 (2) | 8 (8) |
| Total | 148 (62) | 87 (18) | 66 (27) | 67 (7) | 321 (95) | 92 (27) | 57 (38) |
