# Supplementary material for: Biogeographic congruency among bacterial communities from terrestrial sulfidic springs
Source: Front Microbiol. 2014 Sep 8;5:473. doi: 10.3389/fmicb.2014.00473 (PMC4157610; doi:10.3389/fmicb.2014.00473)
Supplement: Supplementary file 2 [file Table2.DOCX]

Supplemental Table 2. Number of sequences (and number of OTUs) for each of the major taxonomic groups from 454 tag pyrosequencing.

| Major Taxonomic Group | Sharon | Richfield | TV1 | TV2 | Sulphur | Palmetto | SCHS |
| --- | --- | --- | --- | --- | --- | --- | --- |
| *Alphaproteobacteria* | 316 (82) | 2 (1) | 233 (40) | 10 (5) | 9 (9) | 17 (11) | 18 (10) |
| *Betaproteobacteria* | 1314 (151) | 2 (1) | 42 (15) | 8 (4) | 11 (6) | 216 (17) | 385 (41) |
| *Deltaproteobacteria* | 308 (59) | 124 (20) | 257 (58) | 86 (12) | 11 (5) | 7 (6) | 954 (105) |
| *Epsilonproteobacteria* | 2268 (256) | 5694 (143) | 1392 (133) | 7837 (126) | 243 (44) | 9 (2) | 138 (14) |
| *Gammaproteobacteria* | 1189 (186) | 2772 (80) | 1039 (161) | 212 (23) | 1602 (47) | 705 (31) | 1285 (69) |
| Acidobacteria | 13 (7) | 0 | 0 | 0 | 1 (1) | 0 | 21 (10) |
| Actinobacteria | 12 (9) | 1 (1) | 4(4) | 0 | 0 | 0 | 19 (9) |
| Aquificae | 0 | 0 | 2 (2) | 0 | 0 | 0 | 2 (2) |
| Bacteroidetes | 1660 (305) | 216 (55) | 1063 (154) | 116 (34) | 84 (12) | 55 (19) | 396 (46) |
| Caldiserica | 0 | 0 | 0 | 0 | 0 | 0 | 1 (1) |
| Chlamydiae | 0 | 0 | 0 | 0 | 0 | 0 | 20 (1) |
| Chlorobi | 15 (6) | 1 (1) | 44 (4) | 265 (10) | 2 (1) | 2 (1) | 110 (11) |
| Chloroflexi | 36 (10) | 20 (9) | 12 (10) | 4 (1) | 5 (3) | 3 (2) | 643 (64) |
| Chrysiogenetes | 0 | 0 | 0 | 0 | 0 | 0 | 1 (1) |
| Cyanobacteria | 0 | 0 | 146 (43) | 3 (3) | 24 (4) | 110 (5) | 1548 (44) |
| Deferribacteres | 0 | 0 | 27 (4) | 1 (1) | 0 | 0 | 17 (6) |
| Deinococcus | 1 (1) | 0 | 0 | 0 | 0 | 0 | 6 (3) |
| Eukaryota | 3 (2) | 0 | 1016 (76) | 9 (4) | 29 (2) | 7 (3) | 0 |
| Fibrobacteres | 0 | 0 | 5 (1) | 1 (1) | 0 | 0 | 0 |
| Firmicutes | 194 (30) | 28 (6) | 377 (41) | 113 (26) | 37 (11) | 1 (1) | 101 (25) |
| Fusobacteria | 1 (1) | 1 (1) | 11 (6) | 0 | 5 (4) | 0 | 0 |
| Gemmatimonadetes | 0 | 0 | 0 | 0 | 0 | 0 | 2 (1) |
| Lentisphaerae | 15 (4) | 1 (1) | 30 (7) | 0 | 0 | 0 | 16 (8) |
| Nitrospira | 0 | 0 | 1 (1) | 0 | 0 | 0 | 0 |
| Planctomycetes | 7 (7) | 0 | 65 (6) | 0 | 2 (2) | 1 (1) | 42 (12) |
| Spriochaetes | 8 (3) | 5 (2) | 3389 (109) | 11 (3) | 0 | 2 (2) | 177 (18) |
| Synergistetes | 0 | 0 | 0 | 0 | 0 | 0 | 2 (1) |
| Tenericutes | 8 (1) | 0 | 1 (1) | 8 (1) | 0 | 0 | 0 |
| Thermodesulfobacteria | 2 (2) | 0 | 2 (2) | 0 | 0 | 0 | 6 (2) |
| Thermotogae | 0 | 0 | 106 (8) | 1 (1) | 0 | 0 | 0 |
| Verrucomicrobia | 74 (25) | 0 | 12985 (218) | 13 (7) | 0 | 12 (5) | 76 (16) |
| Candidate Division BRC1 | 0 | 0 | 0 | 0 | 0 | 0 | 4 (2) |
| Candidate Division OD1 | 2 (1) | 0 | 0 | 0 | 0 | 0 | 20 (7) |
| Candidate Division OP10 | 0 | 0 | 0 | 0 | 0 | 2 (1) | 34 (10) |
| Candidate Division SR1 | 626 (73) | 115 (19) | 34 (8) | 0 | 28 (4) | 8 (3) | 6 (3) |
| Candidate Division TM7 | 3 (1) | 0 | 0 | 0 | 0 | 0 | 0 |
| Unidentified | 197 (62) | 203 (31) | 685 (90) | 110 (17) | 100 (10) | 6 (3) | 273 (31) |
| Totals | 8272 (1284) | 9185 (371) | 22968 (1202) | 8808 (279) | 2193 (165) | 1163 (113) | 6323 (573) |
