# Supplementary material for: Biogeographic congruency among bacterial communities from terrestrial sulfidic springs
Source: Front Microbiol. 2014 Sep 8;5:473. doi: 10.3389/fmicb.2014.00473 (PMC4157610; doi:10.3389/fmicb.2014.00473)
Supplement: Supplementary file 3 [file Table3.DOCX]

Supplemental Table 3. Comparison of dominant groups according to major taxonomic affiliations for Sanger versus 454 tag pyrosequencing from the seven sampled springs.

| **Ranked taxonomic groups (% of the total) from Sanger sequencing** | | | | | | | | | | |
| --- | --- | --- | --- | --- | --- | --- | --- | --- | --- | --- |
| **Sharon Springs** | **Richfield Spring** | | **TV1** | **TV2** | **Sulphur Springs** | | **Palmetto Spring** | **SCHS** |  |  |
| *Epsilonproteobacteria* (27.4) | *Epsilonproteobacteria* (62.0) | | Verrucomicrobia  (56.5) | *Epsilonproteobacteria* (89.0) | *Gammaproteobacteria* (73.0) | | *Gammaproteobacteria*  (60.6) | Cyanobacteria  (24.5) |  |  |
| Bacteroidetes  (20.1) | *Gammaproteobacteria* (30.2) | | Spirochaetes  (14.7) | Chlorobi  (3.0) | *Epsilonproteobacteria* (11.1) | | *Betaproteobacteria* (18.6) | *Gammaproteobacteria*  (20.2) |  |  |
| *Betaproteobacteria* (15.9) | Bacteroidetes  (2.3) | | *Epsilonproteobacteria* (6.1) | Bacteroidetes  (1.3) | Unidentified  (4.6) | | Cyanobacteria  (9.4) | *Deltaproteobacteria* (15.1) |  |  |
| 63.4 | 94.5 | | 77.3 | 93.3 | 88.7 | | 88.6 | 59.8 |  |  |
| **Ranked taxonomic groups (% of the total) from 454 pyrosequencing** | | | | | | | | | | |
| **Sharon Springs** | | **Richfield Spring** | **TV1** | **TV2** | | **Sulphur Springs** | **Palmetto Spring** | **SCHS** | |  |
| Bacteroidetes  (33.1) | | *Gammaproteobacteria* (29.9) | Verrucomicrobia  (40.9) | *Epsilonproteobacteria* (68.6) | | *Gammaproteobacteria* (45.3) | *Gammaproteobacteria* (72.8) | Chloroflexi  (26.3) | |  |
| *Gammaproteobacteria* (24.2) | | Bacteroidetes  (25.3) | Bacteroidetes  (16.7) | Unidentified  (17.9) | | Bacteroidetes  (13.1) | Cyanobacteria  (8.7) | *Gammaproteobacteria* (15.5) | |  |
| *Epsilonproteobacteria* (14.9) | | *Epsilonproteobacteria* (20.7) | *Gammaproteobacteria* (13.6) | Chlorobi  (7.5) | | *Epsilonproteobacteria* (11.5) | Bacteroidetes  (7.6) | Unidentified  (14.0) | |  |
| 72.2^1^ | | 75.9 | 71.2 | 94 | | 69.9 | 89.1 | 55.8 | |  |

^1^Sum of three percentages of the dominant taxonomic groups among all retrieved sequences in site dataset.
